# State of Lunacy in Paris

**Published:** 1854-04-01

**Authors:** 


### STATE OF LUNACY IN PARIS.

A report on the situation of lunatics in the asylums at Paris was presented to the Council General of the Seine in its last session. On the 31st December, 1854, the number of lunatics under treatment was 3182. In all France there were 16,719, which made 1 in every 2123 of the total population; but in Paris and the department of the Seine, the proportion was 1 in every 474. This was owing to the fact that, at Paris, idiots are readily admitted into asylums, in order to prevent them from becoming a spectacle, or being ill treated in the streets; whereas, in the country, great numbers, not being dangerous, are allowed to be at large, and are generally treated kindly by everybody. In fifty-one years the number of lunatics in Paris and the department of the Seine has increased from 946 to 3182. The number of admissions in the course of 1852 was 1509. Amongst them were 454 traders, 149 members of liberal professions, 26 agriculturists, &c. 976 of them belonged to Paris, 182 to the department of the Seine, the rest to different parts of France, and 61 to foreign countries. Amongst the foreigners were 1 Englishman, 21 Belgians, 16 Sardinians, 6 Prussians, and 5 Germans. The number of persons discharged in the course of the year was 849; of cured 556; and of deaths 462. The proportion of deaths was nearly half less than in the ordinary hospitals; and the principal cause of them was paralysis,—that disease having caused 194 of the total. All the lunatics of Paris and the department are not treated in the asylums of Paris; some are maintained in those of the provinces—Blois, Maréville (Meurthe), Arméntires (Nord), &c.—but at the expense of Paris. The expense of each lunatic per day in Paris was 1fr. 50c. for men, and 1fr. 20c. for women; and in the provinces it averaged from 1fr. to 1fr. 25c. The total expense of the year was 1,438,432fr. 78c.; of which 434,065fr. were disbursed in the asylum of Bicêtre, 592,542fr. in that of La Salpétrière, and the rest in the provinces. Part of the expense, however, has to be repaid by the families of the patients, the Prefecture of Police, and the rural communes; and another part by foreign governments,—amongst which governments that of England owes 711fr. 30c., that of Belgium 644fr. 50c., and that of Piedmont 2867fr. 80c. By a law of 1838, two sorts of admissions into lunatic asylums are allowed: one, called "voluntary," is that of non-dangerous lunatics on the demand of their families; the second, called "official," is ordered by the Prefecture of Police, with respect to persons whose maladies are dangerous to themselves or others. Before 1838 the number of official admissions was less than that of voluntary admissions: from 1833 to 1838, for example, the former was 2821, and the latter 4242. But from 1838 to 1851, out of 16,716 admissions, 4163 were voluntary and 12,553 official. Of the 1509 admissions of 1852, 398 were voluntary and 1111 official. Up to the commencement of the nineteenth century, the laws did not occupy themselves with the condition of lunatics.

Confounded with thieves and vagabonds, lunatics were confined in the prisons and hospitals. From a report presented in 1791 to the National Assembly by M. de La Rochefoucauld-Liancourt, it appears that at that time the number of lunatics was 1331. At that period two wards of the Hôtel Dieu were reserved to the curable; but they were often placed three or four together, men and women, in the same bed; the more violent were even bound with chains, and the other patients heard all day long their cries, or witnessed painful scenes.

The incurables were placed at Bicêtre, La Salpêtrière, and the Petites-Maisons (at present Hospice des Ménages.) The cells in which they were confined were only six feet square; light and air were admitted by the door; truckle beds covered with straw, and fastened to the walls, were all they had to sleep on; and water fell from the walls. In 1792, Dr. Pinel, physician of Bicêtre, and afterwards of La Salpêtrière, put an end to this frightful state of things. The creation of the Conseil General des Hospices in 1800 completed his undertaking. Since that time the regard due to misfortune, the care which a suffering being requires, and the protection of individual liberty, have been the sole principles by which legislation relative to lunatics has been guided. The law of the 30th June, 1838, which constitutes the code on the subject, is thus described in the exposé des motifs presented to the Chamber of Peers:—"It is a law of police and of protection with respect to all the citizens, a law of kindness and of guardianship with respect to the insane, and a law of public charity with respect to the unfortunates, whose position and that of their families leaves them without resources." Since that time, however, a good many improvements have been introduced into the treatment of the insane. Spacious and healthy lodgings with boarded floors have been substituted for the old cells; an iron bedstead with excellent bedding, a chair, and a table form the furniture of each room; the number of physicians has been increased, and that of the employés charged to watch over and attend the patients has almost been doubled. The old practice of making the patients dine in their own room has been suppressed, and they take their meals in common in vast refectories, comfortably furnished. The horrible wooden bowls, in which food was formerly served up, have disappeared, and the patients are served in earthenware vessels; each has an iron spoon and fork, a knife and a cup. The meals are preceded and followed by a prayer; at Bicêtre the prayers are chanted in common by the patients. These prudent innovations, by obliging the patients to control themselves in each others' presence, have not a little contributed to the establishment of order, and the respect of rules. Formerly, the clothing allowed was made to last three years. This, however, was carrying economy too far. The state in which the clothes were after such long service, especially those worn by the infirm or the aged, who are generally not remarkable for cleanliness, may be imagined. In 1841 only 13fr. a-year were allowed at La Salpêtrière, and 11fr. at Bicêtre, for the clothing of each patient; but at present nearly double the sum is granted, and the clothes are replaced when unfit for use, without regard to the length of time they have been worn. Work is the most salutary and most efficacious means of action that can be employed in lunatic asylums: it calms when it does not cure, and that constitutes a sensible amelioration. In the two asylums at Bicêtre and La Salpêtrière, 1343 patients work with acuteness which astonishes all visitors; in fact, the silence they observe could certainly not be obtained in the workshops of men in sound health of body and mind. Moreover, work is for these unfortunates a source of profit, and thereby they are able to procure some little comforts not included in the ordinary régime of the establishments. Finally, to amuse the patients and break the monotony of their stay at the hospital, games, singing, gymnastics, drives in the country, &c., are allowed. These experiments have already produced the best results, and all physicians advise that they shall be continued.

